# Evaluating Geographic Distribution and Potential Environmental Risk Factors of Orofacial Cleft Anomalies Utilizing a Statewide Birth Defects Registry

**DOI:** 10.1177/10556656251326443

**Published:** 2025-03-17

**Authors:** Clark Kennedy, Sean Young, Kyle Davis, Erin Weatherford Creighton, Adam Johnson, Larry Hartzell

**Affiliations:** 1 College of Medicine, 12215 University of Arkansas for Medical Sciences, Little Rock, AR, USA; 2 O’Donnell School of Public Health, 12334 University of Texas Southwestern Medical Center, Dallas, TX, USA; 3 Department of Otolaryngology, 12215 University of Arkansas for Medical Sciences, Little Rock, AR, USA

**Keywords:** cleft lip, cleft palate, cleft lip and palate, non-syndromic clefting, facial cleft

## Abstract

**Objective:**

The reported prevalence of cleft lip and palate within the state of Arkansas (10.63 to 12.22 per 10,000 live births) is much higher than the national average. With greater understanding of at-risk populations and risk factors, we can provide more targeted education to improve patient outcomes and potentially reduce the incidence of orofacial clefts.

**Design:**

This is a database review of cleft lip and/or palate patients using data obtained from the Arkansas Reproductive Health Monitoring System (ARHMS) database, a statewide birth defects registry that collects data from 83 regional hospitals.

**Setting:**

Statewide database study.

**Patients:**

Patients diagnosed with cleft lip and/or palate between the years 1993 and 2015 registered in the ARHMS database.

**Main Outcome Measure:**

Prevalence rates of orofacial clefts within each Arkansas county.

**Results:**

A total of 1345 unique patients with a diagnosis of cleft lip and/or palate were identified in the database with an average prevalence of 14.9 per 10,000 live births [95% CI: 14.1–15.7]. Of the 75 counties in Arkansas, 37 counties had prevalence rates higher than the state rate. Three counties had particularly higher prevalence rates of more than 33 per 10,000 live births. On the other hand, 3 counties had very low rates of 5 or less per 10,000 live births.

**Conclusions:**

The rates of orofacial cleft anomalies are not distributed as expected among Arkansas counties. Further exploration is necessary to determine what, if any, environmental factors are at play.

## Introduction

Orofacial cleft anomalies are one of the most common classes of congenital defects, affecting approximately 1 in every 690 births in the United States.^1^ This class encompasses patients with clefts of the hard palate, soft palate, lip, uvula, or a combination of these. In fact, combined cleft lip and palate were found to be the second most common birth condition seen in infants, second only to Trisomy 21.^2^

These anomalies can be due to single-gene mutations, chromosomal abnormalities, environmental agents, or the interplay between genetics and the environment.^3^ The cleft anomaly may be considered syndromic or an isolated defect. In one study, as many as half of the cases of cleft palate were considered isolated, while the number of isolated cases of cleft lip with or without cleft palate was higher at three-fourths.^4^ This difference supports recent studies which have named a distinction in the patterns of inheritance and etiology between cleft palate when compared to cleft lip with or without cleft palate.^5^

These data show a vast majority of patients with orofacial cleft anomalies fall into the category of multifactorial etiology, a combination of genetics and the environment. The list of environmental factors associated with orofacial cleft anomalies is extensive and ranges from known teratogens to factors still being investigated. These factors include maternal alcohol use, maternal cigarette use, certain pesticides, vitamin deficiencies, and pharmacologic agents, including antiepileptic agents,^6^ retinoids,^7^ and corticosteroids.^8^

Orofacial cleft anomalies develop early in pregnancy within the critical weeks of development between weeks 4 and 12.^9^ Most are recognized at the mid-pregnancy anatomy scan, an ultrasound done around 20 weeks, though some can be detected as early as 13 weeks depending on the skill of the sonographer, body type of the patient, fetal positioning, quantity of amniotic fluid, and the type of anomaly.^9,10^ Regardless of the etiology and timing of diagnosis, cleft anomalies are associated with feeding difficulties, speech disorders, recurrent infections, growth and developmental abnormalities, and airway compromise.^11^ Older children and teenagers have increased dental requirements sometimes requiring additional surgeries.^11^ The physical obstacles are coupled with the psychosocial concerns for these patients. Orofacial cleft anomalies bring lifetime health, financial, and quality of life burdens to patients and their families.

The prevalence of cleft lip and palate in the general population has historically been reported as 1 per 1000 births from a study published in 1978.^12^ More recently, the number of patients born with congenital anomalies is increasing at a national level, but the overall national prevalence of orofacial cleft anomalies was found to be lower than previously recorded.^13^ A study published in 2012 found the prevalence of cleft lip and palate in the United States to be 7.75 per 10,000 live births from 2002 to 2006 and the international rate to be 7.94 per 10,000 live births.^13^ However, rates varied from state to state. While the prevalence in some states was found to be lower than the national rate, eg, 2.59 per 10,000 in West Virginia, other states were found to have prevalence rates even higher than those reported in the original studies, such as 21.46 per 10,000 in Maryland.^13^ When looking specifically at Arkansas, the rate of cleft lip with or without cleft palate was 12.22 per 10,000 live births.^13^ This rate is higher than the national rate in both previously cited studies and well above the median when compared to the rates of the other included states.

The purpose of this study was to analyze data from a statewide birth defect registry to identify specific geographic regions and potential environmental risk factors within the state of Arkansas associated with a higher incidence of orofacial cleft anomalies. With this greater understanding of the specific factors and populations at risk, the state would be better equipped to deliver targeted education aimed at improving patient outcomes and quality of life as well as employ prevention techniques aimed at decreasing the incidence of children born with orofacial clefts in Arkansas. Even further, the knowledge gained from this project could be employed to study the birth defects registries of other states with the hopes of bringing increased awareness and prevention efforts at a national level.

## Methods

This study was conducted under the approval of the Institutional Review Board at University of Arkansas (IRB #239682). The data for this project was obtained from the Arkansas Reproductive Health Monitoring System (ARHMS), a statewide birth defects registry founded in 1980 and funded by state legislature starting in 1985. The ARHMS was established as a surveillance system but now also plays a role as a research and prevention organization focusing on gene-environment interactions, epidemiology and surveillance, and health services research and community interventions.^14^ It is one of the oldest active birth defects surveillance systems in the United States.^15^ The ARHMS works with 83 regional hospitals that care for pediatric or obstetric patients. To be entered into the ARHMS database, 3 conditions must be met: the patient must have a birth defect diagnosed by a physician before age 2, ascertained before age 5, and recorded in hospital records.^15^

At the time of institutional review board approval for the study in 2015, data entries from 1980 until the present day were collected from this database. The variables utilized for each infant included: date of birth, date of death (if applicable), and a description of their specific defect. The defects were further described by their date of diagnosis, hospital of diagnosis, and laterality. For each mother, the database recorded their address at the time of delivery, occupation, birthing location, date of birth, age at delivery, and previous reproductive history. For the fathers, the database included their date of birth, race, occupation, and age at the infant's date of birth. For each patient, the family history of birth defects was recorded for first-degree and second-degree relatives. We derived one additional variable, length in delay of presentation, by calculating the difference between the patient's date of birth from their initial diagnosis date. A nonparametric generalized additive model was used to fit a trend line to this data to observe if average differences between the date of birth and diagnosis date have changed over time.

Duplicate patient entries were consolidated using the patients’ first hospital encounter for their cleft diagnosis at one of the 83 regional sites participating in the ARHMS data collection as their diagnosis date. Of over 24 different variables associated with each patient entry, the focus was on patient county and ZIP code, paternal age and occupation, maternal age and occupation, and the time from date of birth to diagnosis date by year. Because of the large variability in parental occupations listed, 17 different occupational categories were identified and named by the research team, and each individual, self-reported occupation was sorted according to best fit.

For spatial analyses, the county of residence at time of birth was used. Using data on live births from 1993 to 2015 from the Arkansas Health Department's Vital Statistics registry, we calculated the orofacial cleft anomalies case rate per 10,000 live births for each county. Data cleaning and analyses were performed, and figures were created using R (v4.1.3), with the packages tidyverse, sf, tigris, and ggpattern.^16^

## Results

The database initially included 2713 entries, including 499 duplicates. Due to incomplete data collection in the years prior to 1993, a further 869 records were excluded. A total of 1345 unique patient cases from the years 1993 to 2015 were identified and included in the analysis. There were approximately 679,612 live births in Arkansas during that time period, giving an overall prevalence of 19.8 per 10,000 live births, although prevalence varied markedly by county.

**Figure 1.**
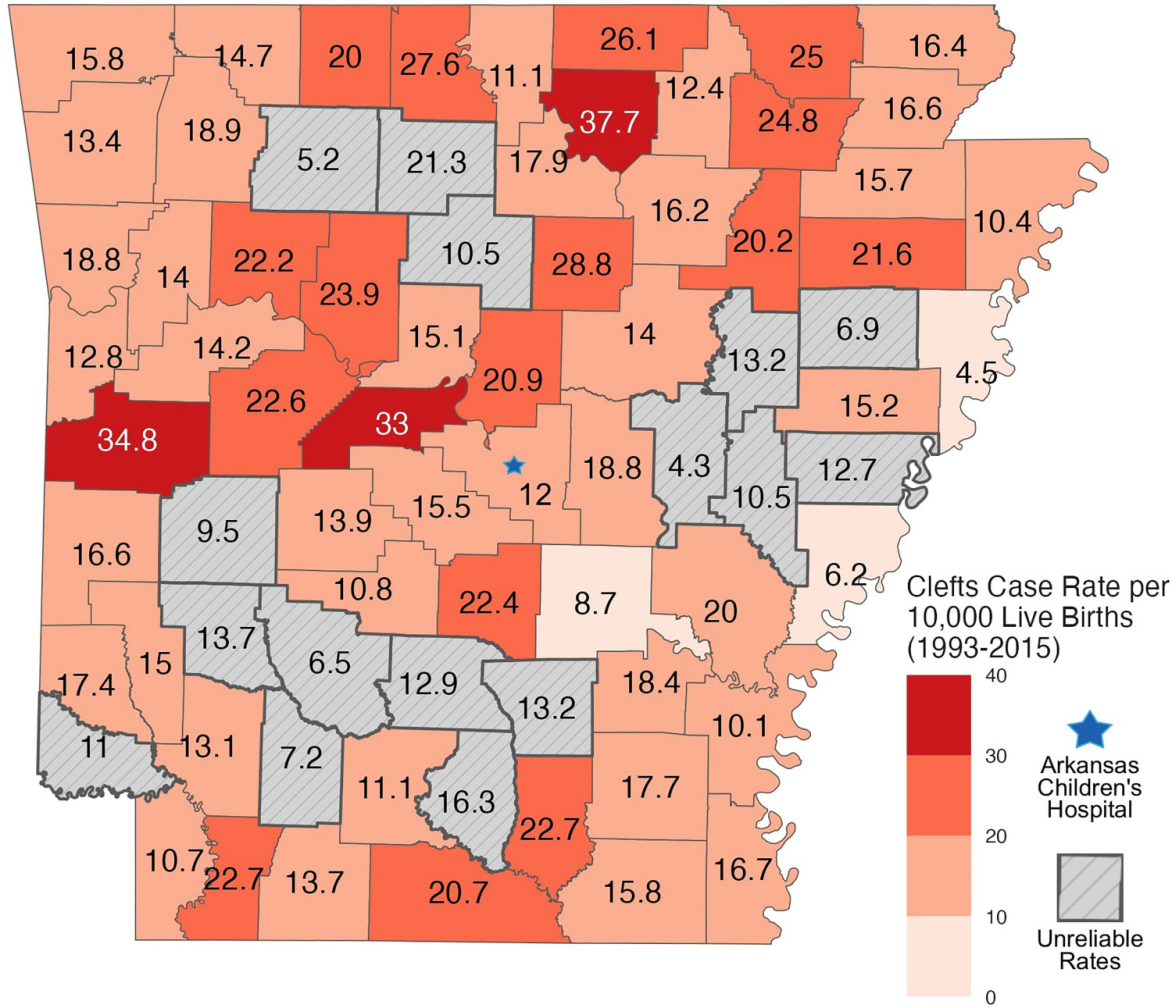
The rate of cleft anomaly cases per 10,000 live births mapped by county from 1993 until 2015.

Figure 1 depicts the rate of orofacial cleft anomalies by county from 1993 until 2015 **(**Figure 1**)**. In order to account for the differences in population and therefore total number of births between counties, county populations and crude birth rates were obtained from the Arkansas Health Department. The 3 most populated counties included Pulaski County, which had 157 total cases, followed by Benton County with 99 cases and Washington County with 88 cases. In contrast to the cumulative number of clefts, the 3 counties with the highest rates of cleft cases per 10,000 live births were Izard County with 38 cases per 10,000, Scott County with 35, and Perry County with 33.

**Figure 2.**
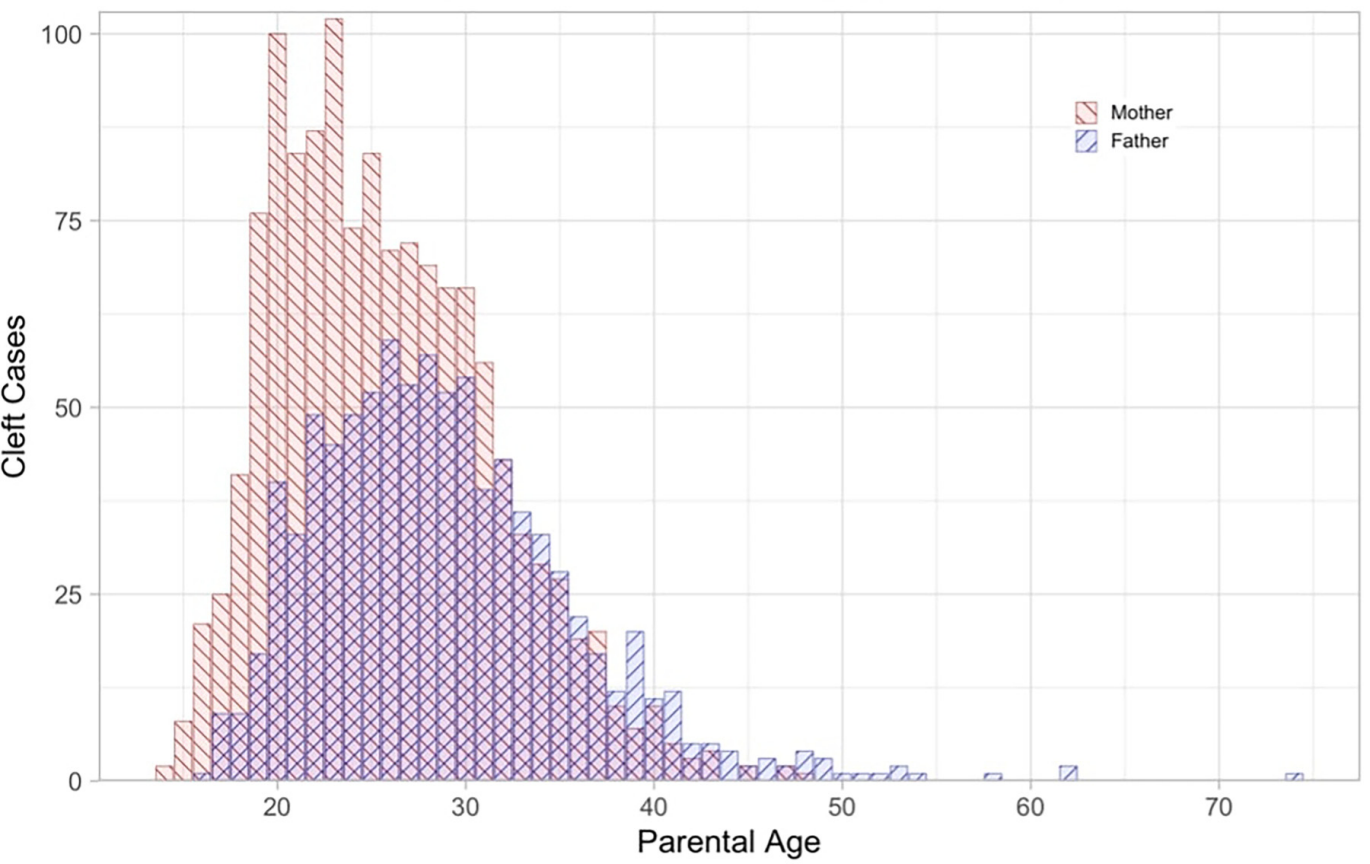
The frequency of cleft anomalies from 1993 through 2015 graphed according to the age of the infant's parents at the time of delivery.

The paternal age associated with the highest number of cleft anomalies was 26 years old. The interquartile range for the paternal age data was found to be 24 to 33 years. The paternal age at the time of birth for cleft cases ranged 58 years from 16 to 74, with a mean of 28.9 (SD: 6.98). In contrast, the maternal age distribution had a higher peak but more limited range. The highest frequency of cleft anomalies occurred at a maternal age of 23 years old. The interquartile range for the maternal age at the time of birth was 21 to 30 years old. The maternal age at the time of birth for cleft cases ranged only 34 years from a low of 14 to a high of 48, with a mean of 25.74 (SD: 5.82) **(**Figure 2**).**

**Figure 3.**
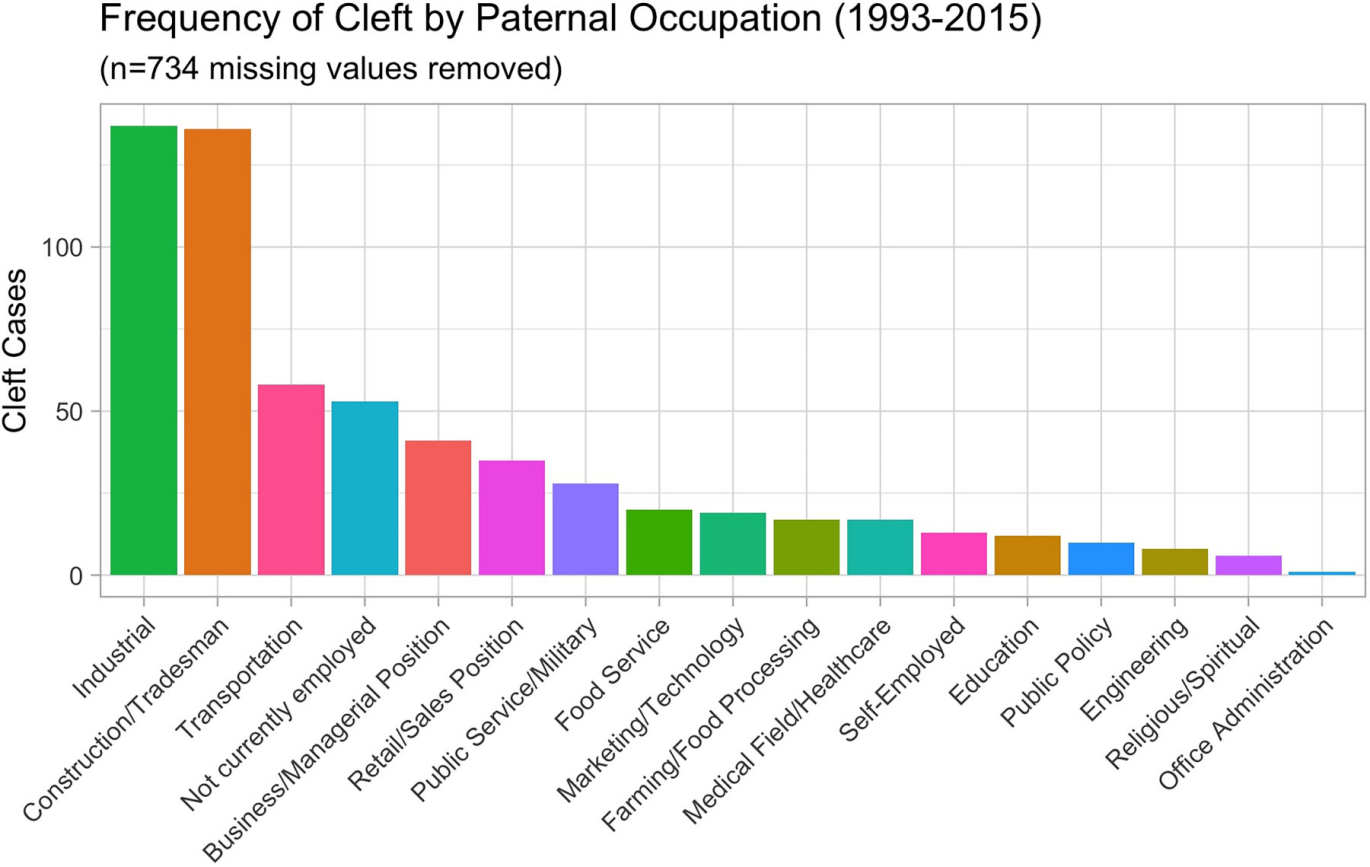
The number of cleft cases from 1993 until 2015 graphed by the category of paternal occupation.

**Figure 4.**
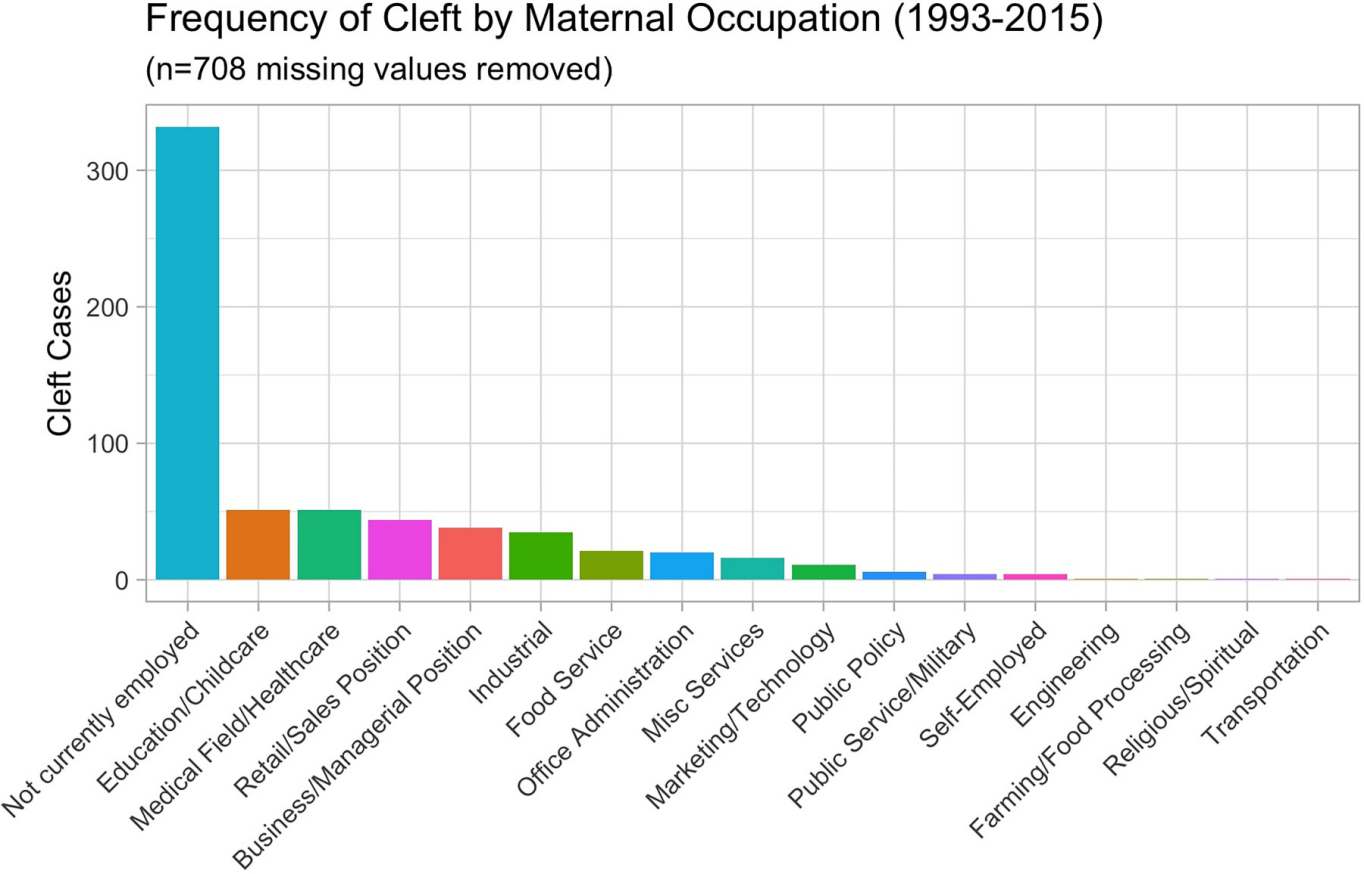
The number of cleft cases in the years 1993 to 2015 graphed by the category of maternal occupation.

The 2 paternal occupations associated with the highest number of cleft anomaly cases were those in industrial (*n* = 137) and construction/tradesman (*n* = 136) lines of work **(**Figure 3**).** When looking at the maternal occupation data, the highest number of cleft cases occurred in those mothers who were not currently employed (*n* = 332). In the mothers who worked outside the home (Figure 4), the categories of education/childcare (*n* = 51) and medical field/healthcare (*n* = 51) were the two with the highest number of cases **(**Figure 4**).**

**Figure 5.**
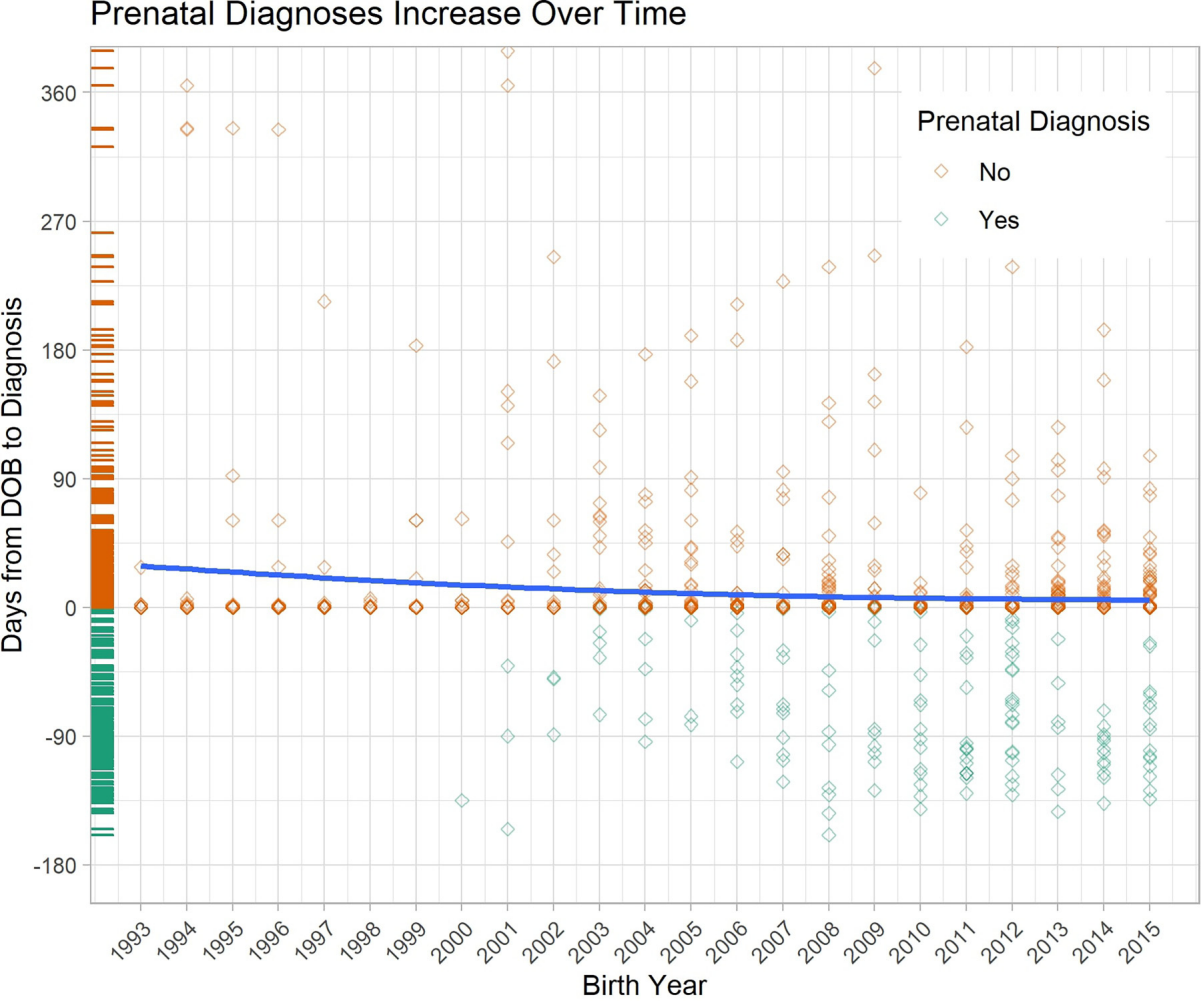
The amount of time from the date of birth until diagnosis of cleft anomaly compared to the birth year of the infant with a trend line depicting the average time between for each year.

The trend line in Figure 5 highlights the gradual but statistically significant trend of an increasing number and proportion of patients being diagnosed prenatally when compared to previous years (*r*^2^ = 0.009, *p* < 0.001). The increasing number of cases with prenatal diagnosis, below Day 0, compared to diagnosis after birth, above Day 0, best shows the strides made toward earlier diagnoses for patients and families **(**Figure 5**).**

## Discussion

When compared to the overall prevalence of orofacial cleft anomalies in the United States at 16.86 per 10,000 births, the prevalence in Arkansas, 19.8 per 10,000 births from 1993 until 2015, is above the national average.^17^ This represents a financial burden for Arkansans, as the estimated lifetime medical cost for one child born with an orofacial cleft anomaly is $100,000.^18^ These children often require multiple surgeries over their lifetime with specialized, coordinated care between multiple disciplines, including otolaryngology, dentistry, speech pathology, audiology, genetics, nursing, and social work.

In this regard, an encouraging finding within this database was the increasing number of early diagnoses for patients and their families between 1993 and 2015 as highlighted by the trend line in Figure 5. This is likely related to 2 factors—improvements in-utero imaging technology and public awareness. The combination of 3D ultrasounds, 4D ultrasounds, and MRI has increased the accuracy and prenatal description of the anomaly regardless of fetal movement and positioning. Four-dimensional ultrasound specifically has shown to be advantageous in parental counseling due to the unique surface rendering mode that allows for better visualization of the surface-level defects of the infant as well as generation of an image of the fetal face that gives parents the best idea of how their infant will look at birth.^10,19–21^ Additionally, there is heightened awareness thanks to both state and nationwide campaigns, including the 2015 decision by the Center for Disease Control and Prevention to name July as National Cleft and Craniofacial Awareness and Prevention Month.^22^ This extra time for patients and families as seen in Figure 5 allows for earlier surgical planning, increased parental understanding and comfort in caring for their infant, and assembly of a multidisciplinary team.

While the overall prevalence in Arkansas was higher than the national average, there were differences within the state as counties varied widely in prevalence with some well below and others almost double that average. The 3 counties with the highest overall prevalence rates included Izard, Scott, and Perry County. These counties are ranked 52, 62, and 60 out of the 75 counties in Arkansas by population, respectively. One limitation was encountered with the initial data obtained from the ARHMS. The database originally included data from 1980 until 2015. However, due to the incomplete entry of data into the database prior to 1993, analysis was performed only on the data from 1993 until 2015. Therefore, 13 years of data was excluded from the analysis. Despite the exclusion, the remaining data captured patients residing in all 75 counties across Arkansas. The ARHMS team monitors all births at the delivering hospitals in the state, primary prenatal diagnostic clinics, and the pediatric specialty hospital to make up over 83 clinical sites.^15^ The geographic distribution of both the hospitals and patients represented in the database and the large area covered by the ARHMS team supports that the majority of the orofacial cleft anomalies during this time period in the state of Arkansas were captured. However, Arkansas is bordered by 6 other states, and it is certainly a possibility that patients residing in border counties sought obstetric or pediatric care in the opposing state. These cases would have been unlikely to be captured within the database.

It is important to consider the locations of the counties in Arkansas, specifically those with higher prevalence rates. Izard county, with the highest prevalence of orofacial cleft anomalies in the state, is 133 miles, or approximately a 2 h 20 min drive time, away from Arkansas Children's Hospital in Little Rock, the only cleft palate and craniofacial repair team in the state. Scott County is located even further at a distance of 138 miles or a 2 h 25 min car trip. Perry County is 53 miles, or 1 h and 6 min away without traffic, which is still at least a 2 h round trip for an appointment. This means families from these counties are required to drive lengthy distances to meet with specialists and receive multidisciplinary care. This represents a major burden—financially, emotionally, and physically—for patients and their families to overcome. While the distance required for travel presents ample opportunities for possible missed appointments and inadequate follow-up, the additional stress this has on the patients’ caretakers is also a substantial factor. The location of the cleft team in Little Rock is already difficult for scheduled in-person visits, but there is an added component of uncertainty for these patients when their child needs care urgently or emergently. The local emergency departments in more rural areas may not be as familiar with caring for these patients who have a wide range of medical complexities and requirements. If the condition is serious, the patient is likely to be transported to Arkansas Children's Hospital—yet another stressor for their family. Access to the level of medical care that these patients need is an obstacle, not only for these 3 counties, but for many counties around Arkansas.

There is a notable discrepancy between these counties with the highest prevalence rates of orofacial cleft anomalies and their population size. The reasons for these differences are not completely clear, and this is an area for future, focused study, however because of the interplay between genetics and the environment in cases of orofacial cleft anomalies, it is important to consider demographic factors along with potential environmental factors. Of the 1345 anomalies between 1993 and 2015, the majority occurred in mothers between the ages of 21 and 30 and fathers between 24 and 33 years. The paternal professions associated with the highest frequency of anomalies were those in the industrial and construction/tradesmen lines of work. The majority of mothers of these infants listed their profession as not currently employed. Of those employed, the 2 most frequently listed were careers in education and healthcare. Another limitation of this study was the variability of maternal and paternal occupations. Because the database only included demographic information on those infants born with cleft anomalies, occupational information for all live births during these years was unavailable, and rates of cleft anomalies by occupation could not be calculated. Calculating the rates would help us understand if a certain parental occupational field is more likely to be associated with cleft anomalies, or if it is just that a larger portion of the workforce in Arkansas has a job in that field. Nonetheless, the role of occupational exposures in the mothers of infants with orofacial cleft anomalies is being investigated.^23^ One study found exposures to fungicides, insecticides, and organic dust associated with an increased risk of cleft palate. Most of the exposures to organic dust were documented in mothers working in agriculture and healthcare. The odds ratio of exposure to organic dust was 1.3 while pesticides was higher at 1.7.^23^ While this particular study did not find an association with cleft anomalies and exposure to solvents, metals, gases, and fumes, occupational and other environmental exposures are important to consider for patients in Arkansas, an agricultural state where 54 out of 75 counties are considered rural.^24^

The impact of maternal smoking on fetal orofacial cleft anomalies is an environmental factor to note when considering Arkansas’ rate of orofacial cleft anomalies compared to that of the nation. A meta-analysis based on 24 different studies found a consistent, statistically significant association between maternal smoking and cleft lip and palate as well as isolated cleft palate. There was an absolute risk of 1 per 450 births, compared to 1 per 600 births in nonsmokers, as well as a 30% increased risk of having a child with cleft lip and palate or a 20% increased risk of having a child with cleft lip.^25^ At the time this research was done in 2004, researchers assumed a 24% prevalence of smoking during pregnancy with more recent data showing a decrease nationwide to 6.0% as of 2019. However, the prevalence of maternal smoking in the state of Arkansas has consistently been higher than the national average, even double that of the national average in 2019, at 12.1%.^26^

Other potential areas of environmental risk for Arkansas to consider include vitamin deficiencies and certain medication interactions. In terms of vitamin deficiencies, there are a mix of findings with multi-vitamin usage. Some studies have found a 50% reduction in the occurrence of cleft lip and palate and a 27% reduction in the occurrence of cleft palate alone.^27^ On the other hand, some studies have yielded no association. However, studies are consistent on the risk of folate deficiency (Vitamin B9) and orofacial clefts among other defects. One study found periconceptional folic acid supplementation halved the risk of cleft lip with or without cleft palate.^28^ There is a known association between medications included in antibiotic, immunosuppressive, and anti-seizure treatment regimens that antagonize folate, or Vitamin B9, receptors and orofacial cleft anomalies. A few others have been linked to cleft anomalies including Isotretinoin, an analogue of Vitamin A, that is listed as a Category X medication and is contraindicated in anyone who may become pregnant. In one particular study, 36 pregnancies were identified with 8 resulting in spontaneous abortion, 1 in stillbirth, and 21 having malformations. Clefting of the secondary palate was noted in the physical exam of a number of these infants.^7^ Corticosteroids are notable with one study finding an odds ratio of 1.7 for cleft lip and palate and 0.5 for cleft palate alone in mothers who indicated corticosteroid use in weeks 4—week 12 after conception when compared to mothers who did not.^8^

These potential environmental factors, parental smoking, vitamin supplementation, and medication reconciliation information were not captured in the ARHMS survey, therefore we do not have the specific data for how these relate to the ARHMS database or the 3 counties in question. However, they are important to consider for the state of Arkansas as a whole when looking to reduce the rate of orofacial cleft anomalies to at least meet the national prevalence. This represents an area for growth in patient counseling and community outreach to increase patient awareness and cleft anomaly mitigation.

With the epicenter for cleft care in Little Rock, it is important to increase community outreach to other parts of the state that may be 2–3 h away. An important component of community outreach in orofacial cleft anomaly education is risk mitigation. Simply saying there is an association is not enough.^25^ To more effectively communicate this risk to expectant parents, the data needs to be clear and direct.

Healthcare providers at the smaller community levels could have a tremendously important role in stressing the importance of folic acid, encouraging smoking cessation, and performing a medication review and subsequent counseling if pregnancy is a possibility or consideration. What future and expectant parents do not know in this situation could hurt their child. Education is key to empowering patients to take these small steps that could lead to a large mitigation of the risk of orofacial cleft anomalies as well as other congenital anomalies.

## Conclusions

The rates of orofacial cleft anomalies in Arkansas are higher than the national average and are not distributed as expected among Arkansas counties. Further exploration is necessary to determine what, if any, environmental factors are at play, and community outreach for risk mitigation and education should be targeted to the entire state, perhaps with additional focus in the identified high-risk counties.

## References

[1] Mai CT Cassell CH Meyer RE , et al. Birth defects data from population-based birth defects surveillance programs in the United States, 2007 to 2011: highlighting orofacial clefts. Birth Defects Res A Clin Mol Teratol. 2014;100(11):895–904. doi:10.1002/bdra.23329 25399767 PMC4631395

[2] Parker SE Mai CT Canfield MA , et al. Updated national birth prevalence estimates for selected birth defects in the United States, 2004-2006. Birth Defects Res A Clin Mol Teratol. 2010;88(12):1008–1016. doi:10.1002/bdra.20735 20878909

[3] Fraser FC . The genetics of cleft lip and palate. Am J Hum Genet. 1970;22(3):336–352. 4910698 PMC1706547

[4] Genisca AE Frías JL Broussard CS , et al. Orofacial clefts in the National Birth Defects Prevention Study, 1997-2004. Am J Med Genet A. 2009;149A(6):1149–1158. doi:10.1002/ajmg.a.32854 19441124 PMC3111146

[5] Beaty TH Ruczinski I Murray JC , et al. Evidence for gene-environment interaction in a genome wide study of nonsyndromic cleft palate. Genet Epidemiol. 2011;35(6):469–478. doi:10.1002/gepi.20595 21618603 PMC3180858

[6] Hernández-Díaz S Werler MM Walker AM Mitchell AA . Folic acid antagonists during pregnancy and the risk of birth defects. N Engl J Med. 2000;343(22):1608–1614. doi:10.1056/nejm200011303432204 11096168

[7] Lammer EJ Chen DT Hoar RM , et al. Retinoic acid embryopathy. N Engl J Med. 1985;313(14):837–841. doi:10.1056/nejm198510033131401 3162101

[8] Carmichael SL Shaw GM Ma C Werler MM Rasmussen SA Lammer EJ . Maternal corticosteroid use and orofacial clefts. Am J Obstet Gynecol. 2007;197(6):585.e1–585.e7. doi:10.1016/j.ajog.2007.05.046 18060943

[9] Arun V Sreejith VP Devarajan AP Gopinath A Sunil M . Psychological effect of prenatal diagnosis of cleft lip and palate: a systematic review. Contemp Clin Dent. 2018;9(2):304–308. doi:10.4103/ccd.ccd_673_17 29875578 PMC5968700

[10] Mayo Clinic. Cleft lip and cleft palate. Published May 22, 2018. Accessed April 5, 2022. https://www.mayoclinic.org/diseases-conditions/cleft-palate/diagnosis-treatment/drc-20370990

[11] Nackashi JA Dixon-Wood V . Health care for children with cleft lip and palate: comprehensive services and infant feeding. In: Dedlow ER ed. Cleft Lip and Palate: From Origin to Treatment. 1st ed. Oxford University Press:303-318.

[12] Habib Z . Genetic counselling and genetics of cleft lip and cleft palate. Obstet Gynecol Surv. 1978;33(7):441–447. doi:10.1097/00006254-197807000-00001 353604

[13] Tanaka SA Mahabir RC Jupiter DC Menezes JM . Updating the epidemiology of cleft lip with or without cleft palate. Plast Reconstr Surg. 2012;129(3):511e–518e. doi:10.1097/prs.0b013e3182402dd1 22374000

[14] National Birth Defects Prevention Network. AR birth defects program. Accessed April 5, 2022. https://www.nbdpn.org/ar_birth_defects_program.php

[15] Smith K . Surveillance: UAMS Fay W. Boozman College of Public Health. Accessed April 5, 2022. https://publichealth.uams.edu/departmentsandunits/centers/arkansas-center-for-excellence-in-birth-defects-research-and-prevention/surveillance/

[16] R Core Team. 2022. R: a language and environment for statistical computing. R Foundation for Statistical Computing, Vienna, Austria. http://www.R-project.org/

[17] Shaye D Liu CC Tollefson TT . Cleft lip and palate. Facial Plast Surg Clin North Am. 2015;23(3):357–372. 26208773 10.1016/j.fsc.2015.04.008

[18] Tolarova MM Al-Kharafi L Boyd C . Pediatric cleft lip and palate. Pediatrics: Surgery. March 2022. https://emedicine.medscape.com/article/995535-overview

[19] Wang LM Leung KY Tang M . Prenatal evaluation of facial clefts by three-dimensional extended imaging. Prenat Diagn. 2007;27(8):722–729. doi:10.1002/pd.1766 17533633

[20] Abramson ZR Peacock ZS Cohen HL Choudhri AF . Radiology of cleft lip and palate: imaging for the prenatal period and throughout life. RadioGraphics. 2015;35(7):2053–2063. doi:10.1148/rg.2015150050 26562237

[21] Merz E Abramowicz JS . 3D/4D Ultrasound in prenatal diagnosis. Clin Obstet Gynecol. 2012;55(1):336–351. doi:10.1097/GRF.0b013e3182446ef7 22343249

[22] Centers for Disease Control and Prevention. Announcement: National Cleft and Craniofacial Awareness and Prevention Month. Morbidity and Mortality Weekly Report (MMWR). July 15, 2011. Accessed May 24, 2024. https://www.cdc.gov/mmwr/preview/mmwrhtml/mm6027a5.htm

[23] Spinder N Bergman JE Boezen HM Vermeulen RC Kromhout H de Walle HE . Maternal occupational exposure and oral clefts in offspring. Environ Health. 2017;16(1):83. doi:10.1186/s12940-017-0294-5 28778209 PMC5545025

[24] Arkansas’s Title V Maternal and Child Health Services Block Grant 2019 Report/2021 Application. HRSA Maternal and Child Health. 2019. Accessed October 12, 2024. https://mchb.tvisdata.hrsa.gov/Narratives/Overview/5e05dd65-f2d2-4bd5-b963-8bd49becf0fe

[25] Little J Cardy A Munger RG . Tobacco smoking and oral clefts: a meta-analysis. Bull World Health Organ. 2004;82(3):213–218. https://www.ncbi.nlm.nih.gov/pmc/articles/PMC2585921/pdf/15112010.pdf 15112010 PMC2585921

[26] Explore smoking during pregnancy in Arkansas: 2021 Health of Women and Children Report. America’s Health Rankings. Accessed April 5, 2022. https://www.americashealthrankings.org/explore/health-of-women-and-children/measure/Smoking_pregnancy/state/AR

[27] Shaw GM Wasserman CR O'Malley CD Tolarova MM Lammer EJ . Risks of orofacial clefts in children born to women using multivitamins containing folic acid periconceptionally. Lancet. 1995;346(8972):393–396. doi:10.1016/s0140-6736(95)92778-6 7623568

[28] Van Rooij IIALM Ocké MC Straatman H Zielhuis GA Merkus HMWM Steegers-Theunissen RPM . Periconceptional folate intake by supplement and food reduces the risk of nonsyndromic cleft lip with or without cleft palate. Prev Med. 2004;39(4):689–694. doi:10.1016/j.ypmed.2004.02.036 15351534

